# RNA-Seq Reveals Transcriptome Changes Following Zika Virus Infection in Fetal Brains in *c-Flip* Knockdown Mice

**DOI:** 10.3390/v16111712

**Published:** 2024-10-31

**Authors:** Ting Xie, Qiqi Chen, Nina Li, Shengze Zhang, Lin Zhu, Shaohui Bai, Haolu Zha, Weijian Tian, Chuming Luo, Nan Wu, Xuan Zou, Shisong Fang, Yuelong Shu, Jianhui Yuan, Ying Jiang, Huanle Luo

**Affiliations:** 1 School of Public Health (Shenzhen), Shenzhen Key Laboratory of Pathogenic Microbes and Biosafety, Shenzhen Campus of Sun Yat-sen University, Shenzhen 518107, Chinatianwj25@mail2.sysu.edu.cn (W.T.); shuylong@mail.sysu.edu.cn (Y.S.); 2 School of Public Health (Shenzhen), Sun Yat-sen University, Guangzhou 510275, China; 3 Key Laboratory of Pathogen Infection Prevention and Control (MOE), State Key Laboratory of Respiratory Health and Multimorbidity, National Institute of Pathogen Biology, Chinese Academy of Medical Sciences & Peking Union Medical College, Beijing 102629, China; 4 Shenzhen Nanshan Center for Disease Control and Prevention, Shenzhen 518054, China; 5 Shenzhen Center for Disease Control and Prevention, Shenzhen 518073, Chinasncdcyjh@szns.gov.cn (J.Y.); 6 Key Laboratory of Tropical Disease Control (Sun Yat-sen University), Ministry of Education, Guangzhou 510080, China

**Keywords:** *c-Flip*, knockdown, fetal head, RNA Seq

## Abstract

The FADD-like interleukin-1β converting enzyme (FLICE)-inhibitory protein (c-FLIP) plays a crucial role in various biological processes, including apoptosis and inflammation. However, the complete transcriptional profile altered by the c-FLIP is not fully understood. Furthermore, the impact of the c-FLIP deficiency on the transcriptome during a Zika virus (ZIKV) infection, which induces apoptosis and inflammation in the central nervous system (CNS), has not yet been elucidated. In this study, we compared transcriptome profiles between wild-type (WT) and the *c-Flip* heterozygous knockout mice (*c-Flip*^+/−^) fetal heads at embryonic day 13.5 from control and PBS-infected WT dams mated with *c-Flip*^+/−^ sires. In the non-infected group, we observed differentially expressed genes (DEGs) mainly involved in embryonic development and neuron development. However, the ZIKV infection significantly altered the transcriptional profile between WT and the *c-Flip*^+/−^ fetal heads. DEGs in pattern recognition receptor (PRR)-related signaling pathways, such as the RIG-I-like receptor signaling pathway and Toll-like receptor signaling pathway, were enriched. Moreover, the DEGs were also enriched in T cells, indicating that the c-FLIP participates in both innate and adaptive immune responses upon viral infection. Furthermore, our observations indicate that DEGs are associated with sensory organ development and eye development, suggesting a potential role for the c-FLIP in ZIKV-induced organ development defects. Overall, we have provided a comprehensive transcriptional profile for the c-FLIP and its modulation during a ZIKV infection.

## 1. Introduction

The FADD-like interleukin-1β converting enzyme (FLICE)-inhibitory protein (c-FLIP) plays a crucial role in numerous diseases, including cancer, diabetes mellitus, rheumatoid arthritis, multiple sclerosis, and Alzheimer’s disease [1,2]. Despite the presence of 13 variants, the c-FLIP is expressed as three distinct proteins in human cells: the 26 kDa short form (c-FLIPS), the 24 kDa form known as c-FLIPR, and the 55 kDa long form (c-FLIPL). The initial animal models have elucidated its critical role in embryo development and regulating death receptor-Fas or TNF-R1 mediated apoptosis [3]. Subsequent studies have affirmed the involvement of c-FLIP in various host biological processes [4,5]. While its primary anti-apoptotic functions, particularly the inhibition of apoptosis cascades such as caspase-8 and caspase-10 activation, have been extensively investigated, the dual role of c-FLIPL, which exhibits a pro-apoptotic function under conditions of low concentration, or exposure to high concentrations of c-FLIPS or c-FLIPR, and intense death receptor stimulation, has also been documented [6,7,8,9]. Moreover, c-FLIPL has been reported to regulate necroptosis mediated by Fas-induced cell death, TLR3, and TNF-induced RIP1/RIP3 [10,11,12]. Additionally, inflammatory signaling, a crucial host response closely related to cell death, is modulated by c-FLIP, acting as a switch between inflammation and cell death by controlling the complex II formation in RIPK1-dependent apoptosis [13]. Notably, the c-FLIP plays an essential role in T cell function, coordinating apoptosis, necroptosis, and autophagy to regulate T cell development and survival [14,15,16]. However, no comprehensive transcriptional profile has been described up until now. Due to its essential role in the host response, several studies have investigated the interaction between c-FLIP and viruses. A recent report has indicated that c-FLIP cleavage can protect mice from SARS-CoV by suppressing the extent of cytokine-induced cell death and apoptosis [17]. Moreover, the c-FLIP can enhance the production of type I interferon (IFN) induced by Coxsackie virus B3 and inhibit virus replication [18]. Conversely, c-FLIP is an essential host factor for the proliferation of the Hepatitis B virus [19]. At the same time, viruses can utilize the c-FLIP to regulate the host response. For example, an infection with Hepatitis C Virus and Herpes Simplex Virus can modulate apoptosis by manipulating the levels of c-FLIP in various cell models [20,21]. Overall, these results indicate a varying role for the c-FLIP during different virus infections, suggesting the need for further exploration.

Zika virus (ZIKV), belonging to the genus *Flavivirus* and the family Flaviviridae, is an icosahedral enveloped arbovirus with a positive-sense, single-stranded RNA genome of approximately 11 kb. The ZIKV can be transmitted to humans through the bite of infected *Aedes aegypti* and *Aedes albopictus* (Asian tiger mosquito) [22,23]. Additionally, the ZIKV can be vertically transmitted from infected women to their embryos during pregnancy, potentially causing a range of adverse fetal outcomes collectively termed congenital Zika syndrome (CZS) [23]. Previous studies have shown that the host E3 ubiquitin ligase Pellino1 promotes cell death in human placental cells during a ZIKV infection [24], while also modulating apoptosis by influencing the c-FLIP levels, a vital factor in regulating the host immune responses [25]. Nevertheless, the precise role of the c-FLIP during a ZIKV infection has not been fully clarified and requires further exploration. Recently, we discovered the facilitative role of the c-FLIP in ZIKV pathogenesis using *c-Flip* heterozygous knockout mice (*c-Flip*^+/−^) [26]. However, the transcriptional implications of the c-FLIP during a ZIKV infection remain incompletely understood. RNA sequencing (RNA-seq) was performed on fetal heads obtained from wild-type (WT) dams subjected to either PBS treatment or a ZIKV infection. The dams were paired with *c-Flip*^+/−^ sires, leading to the presence of both WT and *c-Flip*^+/−^ fetuses (unpublished manuscript). While substantial data were acquired, a comprehensive analysis has not yet been undertaken. In this subsequent study, we characterized the comprehensive set of differentially expressed genes (DEGs) resulting from the knockdown of the *c-Flip* in the embryonic days (E) 13.5 fetal heads. Additionally, we identified the transcriptional profile of ZIKV-induced DEGs in the context of *c-Flip* deficiency, aiming for a better understanding of its role in viral infection.

## 2. Materials and Methods

### 2.1. Virus

The ZIKV strain GZ01 (GeneBank accession no: KU820898) was originally isolated from a Chinese patient, who returned from Venezuela in 2016, and was generously provided by Prof. Jincun Zhao (Guangzhou Medical University, Guangzhou, Guangdong province, China). Viral stocks were generated by infecting African green monkey (Vero) cells (MOI = 0.01) and harvesting supernatants at 96 h post infection (hpi). Viral stocks were stored in aliquots at −80 °C.

### 2.2. Plaque-Forming Assay

Viral titers of stocks were determined via a plaque assay on the Vero cell line (ATCC, CCR-81), which was generously provided by Prof. Caijun Sun from the Sun Yat-sen University and cultured in Dulbecco’s modified Eagle’s medium (DMEM, Biosharp, Hefei, China, C11995500BT) supplemented with 5% heat-inactivated FBS (FBS, Biosharp, FB25015) and 100 U/mL Penicillin-Streptomycin (PS, ThermoFisher, Waltham, MA, USA, 15140122) and cultured at 37 °C in a 5% CO_2_ atmosphere. Viral titers were determined by performing a plaque assay on a monolayer of Vero cells, following the previously described protocol [27].

### 2.3. Murine Models of ZIKV Infection

The *c-Flip*^+/−^ mice (on a C57BL/6 background) were generated using CRISPR/Cas9 by the Gempharmatech company (Nanjing, China). Female wild type mice aged 8 to 10 weeks and male *c-Flip*^+/−^ mice aged 10 to 12 weeks were set up for timed-mating and at E5.5 pregnant mice were inoculated with 2 mg of anti-IFNAR antibody (MAR1-5A3, Leinco Technologies). At E6.5, the mice were infected intraperitoneally (i.p.) with 200 µL of 1 × 10^6^ plaque forming unit (PFU), and the mock-infected mice were injected with 200 µL PBS as a control. The mice in each group were observed daily for the signs of disease and death for 7 days post infection (dpi). They were sacrificed at E13.5, and the fetal heads were collected for a viral load study and transcriptome analysis. Four repetitions were set for each group.

### 2.4. RNA Extraction Library Construction and Sequencing

The total RNA from the fetal head tissue was extracted and purified using TRIzol reagent following the manufacturer’s procedure. After the total RNA was extracted, the mRNA was purified from the total RNA (1 μg) using Dynabeads Oligo (dT) 25-61005 (Thermo Fisher, Waltham, MA, USA) with two rounds of purification. Then the cleaved RNA fragments were reverse-transcribed to create the cDNA by SuperScript™ II Reverse Transcriptase (Invitrogen, Waltham, MA, USA). Subsequently, the sequencing of the cDNA was performed on an Illumina NovaseqTM 6000 platform by LC-Bio Technologies (Hangzhou, China). As shown in Supplementary Table S1, the statistics of the Illumina RNA-Seq reads in the normal and transgenic mice fetal head libraries were mapped to the mice reference genome with a good sequence quality. StringTie was used to perform the expression level for the mRNAs by calculating the Fragments Per Kilobase of exon model per Million mapped fragments (FPKM). Differentially expressed mRNA with fold change >2 or fold change <0.5 was selected and with a parametric F-test comparing nested linear models (*p*-value < 0.05) by R package edgeR.

RNA-sequencing data from this study have been deposited in the NCBI Gene Expression Omnibus (GEO) database (http://www.ncbi.nlm.nih.gov/geo/; accessed on 10 October 2024) under accession number GSE254990, and raw sequence data deposited at the NCBI Sequence Read Archive (SRA) database (http://www.ncbi.nlm.nih.gov/sra; accessed on 30 August 2024) under accession number PRJNA1072959.

### 2.5. Quantitative Polymerase Chain Reaction (qPCR)

The samples were re-suspended in TRIzol reagent (Life Technologies, Invitrogen, Carlsbad, CA, USA) for RNA extraction and cDNA was synthesized by using a qScript cDNA synthesis kit (Bio-Rad, Hercules, CA, USA, 1708891). The sequences of the primer set for *GAPDH*, *NS5*, *Ntrk1*, *Spp1*, *Hoxd3*, *Nefl*, *Rag1*, *RSad2*, *Pagr1a*, *Cryaa*, *Crygd*, *Olig2*, *Myh7b,* and *Nkx2-1* were listed in Supplementary Table S2. The sequences of the primer set for mouse *GAPDH* and ZIKV *NS5* were described previously [24,28]. The specific primer of *Cryaa* (id: 113931666c1) was obtained from the PrimerBank database (https://pga.mgh.harvard.edu/primerbank/; accessed on 28 January 2024) [29]. And other primers were designed on the NCBI website (https://www.ncbi.nlm.nih.gov; accessed on 30 January 2024). The qPCR was performed in the CFX96 real-time PCR system (Bio-Rad, Hercules, CA, USA) with ChamQ Universal SYBR qPCR Master Mix (Vazyme, Nanjing, China, Q711-02) reagent. All the primers were synthesized by Sangon Biotechnology (Shanghai, China).

### 2.6. Data Analysis

The volcano plot, Venn diagrams, Gene Ontology (GO) and Kyoto encyclopedia of Genes and Genomes (KEGG) pathway enrichment analysis of the DEGs were performed and visualized using the OmicStudio tools (https://www.omicstudio.cn/index/; accessed on 7 January 2024) with the clusters Profiler R package on the OmicStudio platform. The protein–protein interaction (PPI) network of DEG was obtained from the STRING database (https://cn.string-db.org; accessed on 20 November 2023) [30] and visualized using Cytoscape software (version 3.8.1) [31]. The hub genes were identified using the Cytoscape plug-in cytoHubba (version 0.1) based on the MCC calculation method [32]. Individual networks were generated from DEGs associated with GO and KEGG categories related to pathways of our interest using an online search with the GeneMANIA website (http://genemania.org; accessed on 28 December 2023) [33]. The z-scores of GO and KEGG analysis were calculated using the R package GOplot v.1.0.2 in R-4.3.2 [34]. Then the results of the GO and KEGG analysis were plotted using R package ggplot2 v3.4.4, paletteer v1.6.0, and ggrepel v0.9.5 [35]. The BiNGO plug-in in Cytoscape was used to perform functional and pathway enrichment analysis [36].

## 3. Results

### 3.1. c-Flip Deficiency Alters the Transcriptomic Profile in Murine Fetal Heads

**Figure 1:**
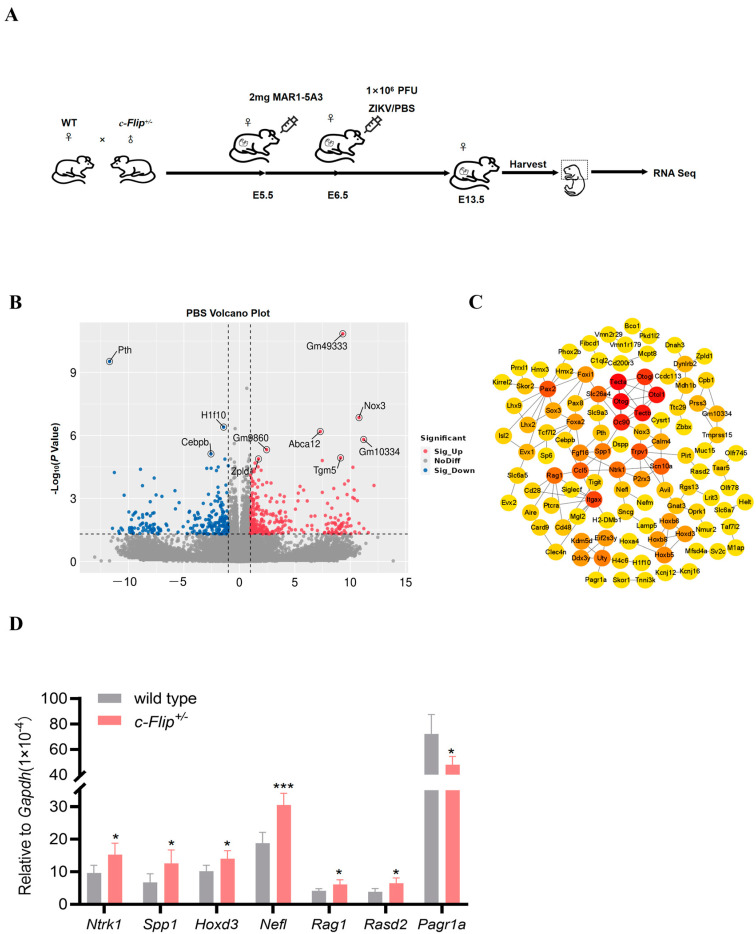
(**A**) A schematic diagram of the experimental design. The wild-type (WT) dams mated with *c-Flip* heterozygous knockout mice (*c-Flip^+/−^*) sires were treated with 2 mg MAR1-5A3 on the day prior to the infection and then i.p. inoculated on E6.5 with PBS or 1 × 10^6^ plaque forming unit (PFU) of Zika virus (ZIKV). The WT and *c-Flip*^+/−^ fetal heads were harvested on E13.5 and utilized for RNA sequencing. (**B**) Volcano plot of differentially expressed genes (DEGs) from the *c-Flip*^+/−^ fetal heads compared with the WT fetal heads of fetuses delivered by PBS-treated WT dams. The red dots illustrate up-regulated genes, the blue dots represent down-regulated genes, and the grey dots show insignificant genes. (**C**) The total hub genes from the *c-Flip*^+/−^ fetal heads compared with the WT fetal heads of fetuses delivered by PBS-treated WT pregnant dams were screened by the CytoHubba Maximum Clique Centrality (MCC) algorithm in Cytoscape. The number of connections is reflected in the degree value of the node, where the intensity of the node’s color indicates a higher degree value and highlights the importance of a specific node in this network. (**D**) The hub genes were assessed by quantitative real-time polymerase chain reaction (RT-qPCR) in the fetal heads. The data are presented as the mean ± SD of *n* = 5. The significance of the differences was determined using the two-tailed Student’s *t*-test, with * *p* < 0.05 and *** *p* < 0.001.

We initiated our study by analyzing the RNA-Seq data derived from the fetal heads at E13.5 of WT dams mated with *c-Flip*^+/−^ sires to identify DEGs resulting from the *c-Flip* knockdown (Figure 1A). The DEGs were determined using the edgeR package with criteria of log2 fold-change (FC) >1 or <−1 and a false discovery rate (FDR) *p*-value < 0.05. As illustrated in Figure 1B, we discovered 528 DEGs in *c-Flip*^+/−^ fetal heads compared to WT fetal heads, comprising 295 up-regulated DEGs and 233 down-regulated DEGs. Subsequently, we constructed a protein–protein interaction (PPI) network using the Maximum Clique Centrality (MCC) algorithm, implemented through CytoHubba in Cytoscape. Within this network, numerous central genes associated with fetal development or the nervous system were identified (Figure 1C). Subsequently, we validated the upregulation of the mRNA levels of *Ntrk1*, *Spp1*, *Hoxd3*, *Nefl*, *Rag1,* and *Rasd2*, as well as the downregulation of the *Pagr1a* mRNA level in the *c-Flip*^+/−^ fetal heads compared to the WT fetal heads using RT-qPCR (Figure 1D).

### 3.2. c-Flip Deficiency Affects Various Biological Processes Associated with Fetal Development

**Figure 2:**
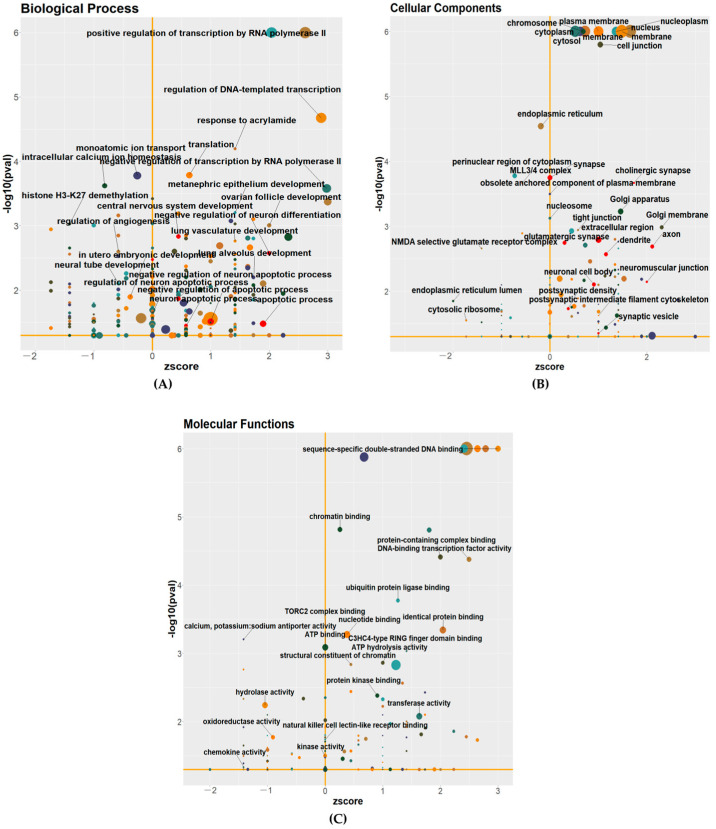
(**A**–**C**) Functional categorization of DEGs from *c-Flip*^+/−^ fetal heads compared with WT fetal heads of fetuses delivered by PBS-treated WT dams were assigned to three Gene Ontology (GO) classes: biological process (**A**), cellular component (**B**), and molecular functions (**C**). The size of the bubbles in the plots is proportionate to the number of associated genes. The significantly enriched categories of our interest are marked or labeled.

To deepen our comprehension of the functional implications stemming from the DEGs in the *c-Flip*^+/−^ fetal heads, we conducted a GO functional enrichment analysis. This analysis aimed to identify alterations in the biological processes, molecular functions, and cellular components. Unexpectedly, we identified the DEGs associated with the biological processes related to apoptosis, including terms such as “apoptotic process” and “negative regulation of the apoptotic process” (Figure 2A). As we selected the E13.5 embryos, we observed that the DEGs were enriched in the biological processes of “in utero embryonic process” and “central nervous system development”. This enrichment may be associated with the DEGs related to neuronal survival, including processes such as “neuron apoptotic process”, “regulation of neuron apoptotic process”, and “negative regulation of neuron apoptotic process” (Figure 2A). Additionally, we observed that the DEGs were associated with several critical neuron-related components, including “axon”, “synapse”, “dendrite”, “neuronal cell body”, “glutamatergic synapse”, and “cholinergic synapse”, as part of the cellular component (Figure 2B). In terms of the molecular functions, our analysis revealed that the DEGs were associated with functions related to immune response, including “chemokine activity” and “natural killer cell lectin-like receptor binding” (Figure 2C). These results indicate that the c-FLIP is involved in neuron system development and the host immune response.

**Figure 3:**
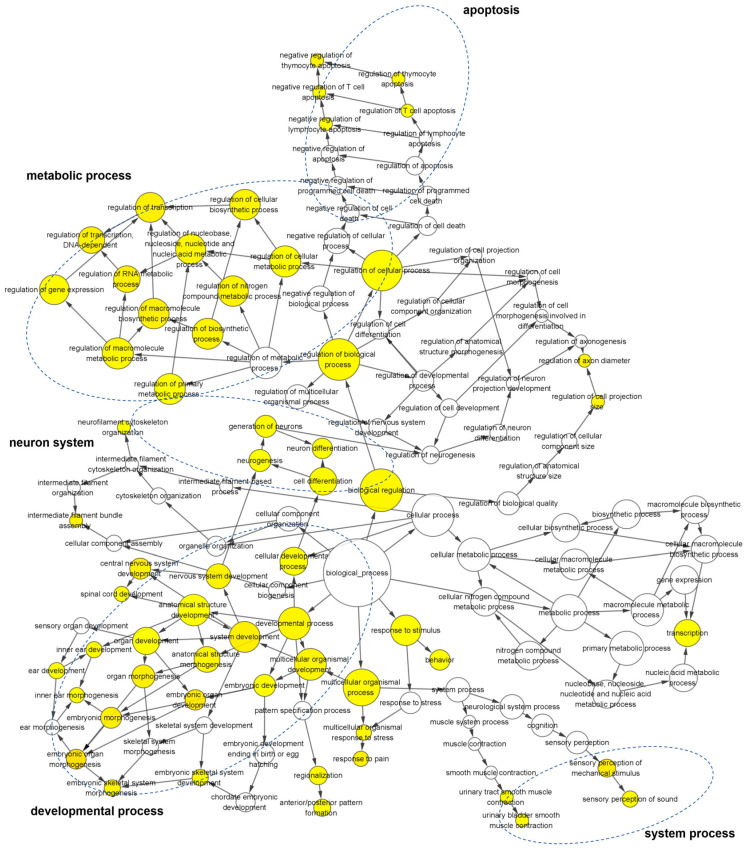
The gene ontology network of the enriched biological process based on the DEGs from the *c-Flip*^+/−^ fetal heads compared with the WT fetal heads of fetuses delivered by the PBS-treated WT dams in the sub network is displayed using the BinGO plug-in for Cytoscape. The color depth of the nodes refers to the corrected *p*-value of the ontologies. The yellow color indicates the highly enriched processes. The deeper color indicates a higher degree of enrichment. The size of the nodes refers to the number of genes that are involved in the ontologies. The larger size indicates more genes.

To unveil the interconnections among significantly altered biological processes, we employed the BiNGO plug-in in Cytoscape to construct interaction networks (Figure 3). Consistent with the aforementioned findings, a notable enrichment was observed in the biological processes related to embryonic development, including “embryonic organ morphogenesis”, “embryonic organ development”, and “embryonic skeletal development”. Noteworthy among the DEGs were those associated with the ear development processes, such as “ear development”, “inner ear development”, and “inner ear morphogenesis”, suggesting a potential role for *c-Flip* in ear development. Furthermore, the deficiency of *c-Flip* resulted in the enrichment of DEGs in central nervous system (CNS) related biological processes, encompassing “central nervous system development”, “nervous system development”, “spinal cord development”, “generation of neurons”, “neuron differentiation”, and “neurogenesis”. These results collectively underscore the intricate involvement of *c-Flip* neuron system development.

### 3.3. The Functional Classification of the DEGs Was Altered with the Reduced Level of the c-Flip

**Figure 4:**
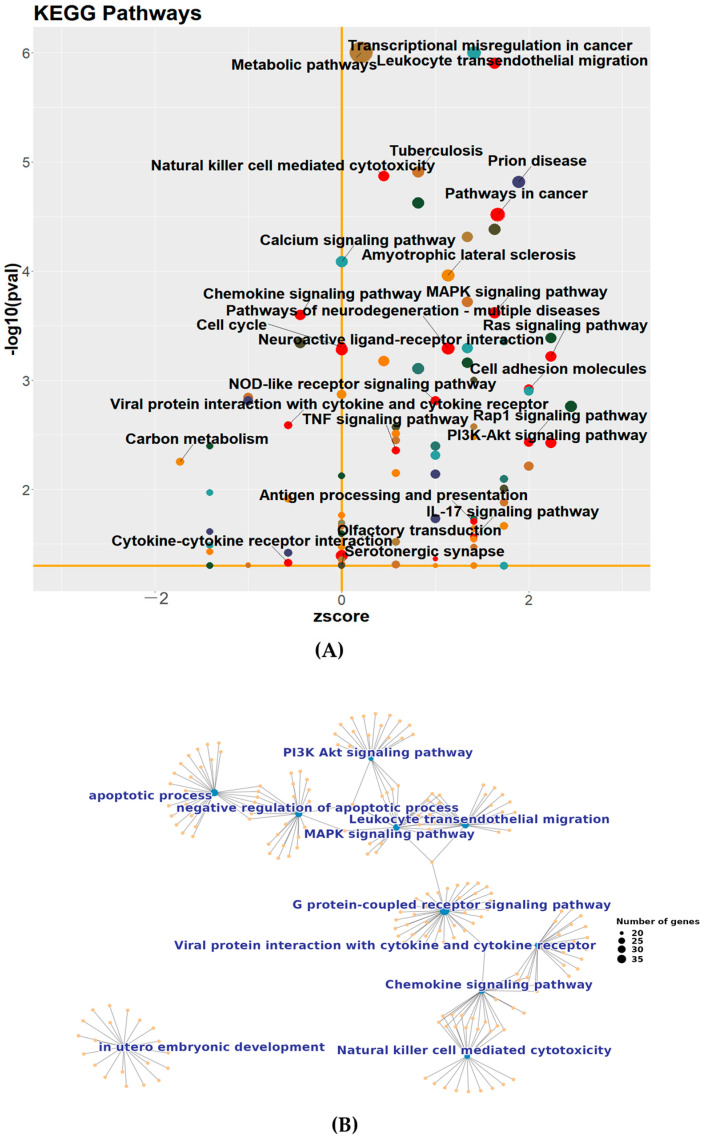
(**A**) The Kyoto encyclopedia of Genes and Genomes (KEGG) pathway enrichment analysis of DEGs in *c-Flip*^+/−^ fetal heads compared with WT fetal heads of fetuses delivered by PBS-treated WT dams. The size of the bubbles in the plots is proportionate to the number of associated genes. The significantly enriched categories of our interest are marked or labeled. (**B**) The enrichment network plot of several pathways in a GO and KEGG analysis of the DEGs from the *c-Flip*^+/−^ fetal heads compared with the WT fetal heads of fetuses delivered by PBS-treated WT dams in the subnetwork are displayed. The size of the blue point on the plots is proportionate to the number of associated genes, and the orange point means individual genes.

Further KEGG analysis revealed that the *c-Flip* knockdown resulted in DEGs enriched in various cell death-related pathways, including the “P13K-Akt signaling pathway”, “MAPK signaling pathway”, and “Ras signaling pathway” (Figure 4A). Notably, the DEGs demonstrated connections with inflammation-related pathways, encompassing “chemokine signaling pathways”, “natural killer cell-mediated cytotoxicity”, “TNF signaling pathway”, “IL-17 signaling pathway”, and “Leukocyte transendothelial migration” (Figure 4A). As our analysis revealed shared genes within GO categories associated with apoptosis and the immune system, we further explored these connections by reconstructing the gene interaction networks for several biological pathways from both GO and KEGG categories using GeneMANIA (http://genemania.org/search/mus-musculus/; accessed on 28 December 2023), as illustrated in Supplementary Figure S1. After merging these networks, we explored the resulting topology. The merged network consisted of ten clusters, with nine displaying interconnections, indicating cross-talk among all affected processes, except for “in utero embryonic development” (Figure 4B). In addition to the tight interconnection between genes associated with the “apoptotic process” and “negative regulation of the apoptotic process”, the “MAPK signaling pathway” and “leukocyte transendothelial migration” shared several nodes and were linked with “apoptosis-related genes” (Figure 4B). The immune response-related pathways, including the “chemokine signaling pathway”, “natural killer cell-mediated cytotoxicity”, and “viral protein interaction with cytokine and cytokine receptor”, were interconnected (Figure 4B). These connections suggest that c-FLIP may play a key role in influencing multiple biological processes.

### 3.4. The Deficiency of c-Flip Impact the Transcriptome of Both Innate and Adaptive Immune Responses in the Fetal Heads During ZIKV Infection

**Figure 5:**
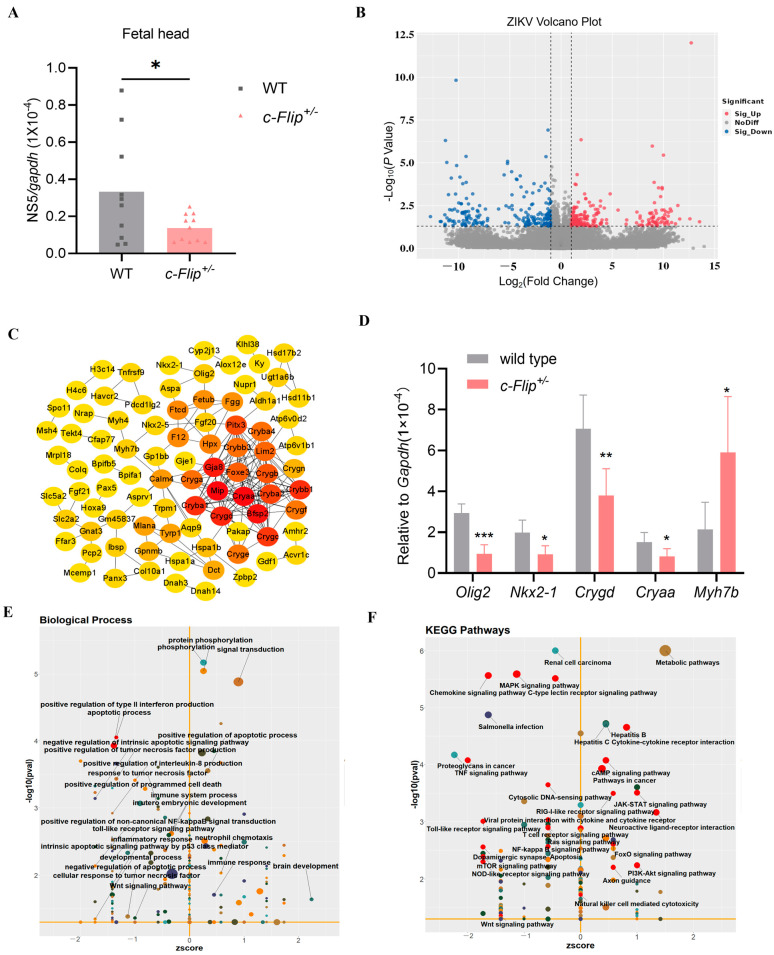
(**A**) ZIKV mRNA in WT and *c-Flip*^+/−^ fetal heads of fetuses delivered from ZIKV-infected WT dams was measured by qPCR. Data are collected from 3–4 pregnant dams per group and analyzed by unpaired Student’s *t* test. (**B**) Volcano plot of DEGs from *c-Flip*^+/−^ fetal heads compared with WT fetal heads of fetuses delivered by ZIKV-infected WT dams. Red dots illustrate up-regulated genes, blue dots represent down-regulated genes, and grey dots show insignificant genes. (**C**) The total hub genes from the *c-Flip*^+/−^ fetal heads compared with the WT fetal heads of fetuses delivered by the ZIKV-infected WT dams were screened by the CytoHubba MCC algorithm in Cytoscape. The number of connections is reflected in the degree value of the node, where the intensity of the node’s color indicates a higher degree value and highlights the importance of a specific node in this network. (**D**) The hub genes were assessed by RT-qPCR in the fetal heads. The data are presented as the mean ± SD of *n* = 5. The significance of the differences was determined using the unpaired Student’s *t* test. (**E**) The functional categorization of the DEGs from the *c-Flip*^+/−^ fetal heads compared with the WT fetal heads of the fetuses delivered by ZIKV-infected WT dams were assigned to one GO class: biological process. The size of the bubbles in the plots is proportionate to the number of the associated genes. The significantly enriched categories of our interest are marked or labeled. (**F**) The KEGG pathway enrichment analysis of the DEGs from the *c-Flip*^+/−^ fetal heads compared with the WT fetal heads of the fetuses delivered by ZIKV-infected WT dams. The size of the bubbles in the plots is proportionate to the number of associated genes. The significantly enriched categories of our interest are marked or labeled. The data are presented as the mean ± SD of *n* = 5. * *p* < 0.05, ** *p* < 0.01, *** *p* < 0.001 compared to control group.

Subsequently, we analyzed the RNA sequencing data from both the WT and *c-Flip*^+/−^ fetal heads born to the same ZIKV-infected WT dams mated with *c-Flip*^+/−^ sires to comprehensively assess the transcriptional profile alterations between the WT and *c-Flip*^+/−^ during ZIKV vertical transmission. We have observed higher ZIKV RNA levels in the WT fetal heads compared with the *c-Flip*^+/−^ heads (Figure 5A). A total of 391 DEGs were identified, comprising 191 up-regulated genes and 200 down-regulated genes (Figure 5B). Notably, the majority of DEGs enriched in the ZIKV-infected group differed from the non-infected group (Figure S2), indicating that the ZIKV infection has profoundly altered the transcriptional profile in both the WT and *c-Flip*^+/−^ fetal heads. The downregulation of the expression of hub genes *Cryaa, Crygd, Nkx2-1,* and *Pax5*, along with the upregulation of *Myh7b* was validated through qPCR (Figure 5C,D).

**Figure 6:**
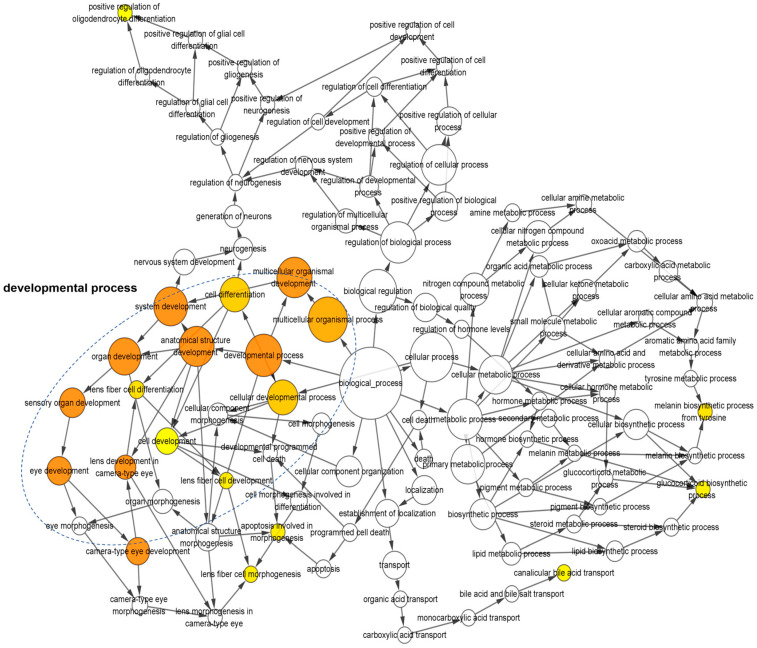
The gene ontology network of the enriched biological process based on the DEGs from the *c-Flip*^+/−^ fetal heads compared with the WT fetal heads of the fetuses delivered by ZIKV-infected WT dams in the sub network is displayed using the BinGO plug-in for Cytoscape. The color depth of nodes refers to the corrected *p*-value of the ontologies. The yellow color indicates the highly enriched processes. The deeper color indicates a higher degree of enrichment. The size of the nodes refers to the number of genes that are involved in the ontologies. The larger size indicates more genes.

Further GO functional enrichment analysis indicates that the majority of the DEGs are associated with anti-viral immune response signaling, including “TNFα signaling”, “type II IFN signaling”, “NF-κB signaling”, and the “Toll-like receptor signaling pathway”, in addition to the c-FLIP-induced cell death-related signaling (Figure 5E). These results suggest that the c-FLIP was deeply involved in the host immune response during the ZIKV infection. Consistent with the GO analysis, further KEGG pathway enrichment demonstrated that the DEGs were associated with classic anti-viral immune response signaling, particularly the pattern recognition receptor (PRR)-related signaling pathways, such as “RIG-I-like receptor signaling pathway”, “Toll-like receptor signaling pathway”, “cytosolic DNA sensing pathway”, and “NOD-like receptor signaling pathway” (Figure 5F). This suggests the involvement of the c-FLIP in the innate immune response. Additionally, we observed connections of DEGs with the C-type lectin receptor signaling pathway and T cell receptor signaling pathway, indicating an important role for the c-FLIP in regulating adaptive immune responses (Figure 5F). Notably, we observed a connection among the DEGs involved in the biological processes such as “anatomical structural development”, “sensory organ development”, and “eye development” (Figure 6). Overall, these results suggest that the c-FLIP participates in the anti-ZIKV infection response and may play a potential role in ZIKV-induced organ development defects.

## 4. Discussion

Numerous in vitro studies have underscored the pivotal role of the c-FLIP in apoptosis induced by death receptors. There is growing evidence supporting the idea that the c-FLIP plays diverse roles in cellular homeostasis, exerting distinct influences on the same pathways depending on its expression level and predominant isoform [9,37]. In vivo studies have faced limitations, as the complete deficiency of the c-FLIP leads to fetal death past E 10.5 [3]. Consequently, conditional or heterozygous knockout mice have been developed for in vitro studies [14,38]. In this study, we utilized heterozygous knockout mice to comprehensively explore the role of the c-FLIP in the fetal heads and described the altered transcriptional profile induced by ZIKV.

Choosing the *c-Flip*^+/−^ fetal heads at E13.5, we observed DEGs enriched in embryo development and CNS development. Yeh et al. reported no abnormalities in the gross examination of the *c-Flip*^+/−^ mice [3]. In our study, we found no size or weight differences between the WT and *c-Flip*^+/−^ fetuses at E13.5 [26]. However, despite the absence of observable morphological differences, we identified an altered transcriptional profile involving multiple critical signaling pathways related to fetal development. Consistent with prior studies, we have observed that the knockdown of the c-FLIP induced-DEGs enriched in well-studied biological processes, such as apoptosis and the regulation of T cell apoptosis. However, the role of the c-FLIP in CNS development is not widely recognized, despite our observation of the enriched DEGs in neuron differentiation and CNS development. A study has demonstrated that the c-FLIP can protect oligodendrocytes from neuroinflammation, which is related to degenerative central nervous system diseases [39], suggesting that the c-FLIP may indirectly regulate CNS development through other signaling pathways. Nevertheless, we cannot ignore the possibility that the c-FLIP may be directly involved, an aspect that requires further in-depth investigation.

We further investigated the role of the c-FLIP during ZIKV-induced congenital zika syndrome and found the DEGs associated with multiple classic innate immune responses against virus infection. One possible explanation is the varied viral load caused by the c-FLIP deficiency. Nevertheless, the enrichment of the DEGs in the innate immune system suggests a critical role for the c-FLIP in the innate immune response, despite potential cross-connections within the host’s biological processes. Notably, we have found that the c-FLIP is associated with sensory organs, especially the eye. Previous studies have shown that the eye is a target organ for the ZIKV [40,41], but the detailed mechanism is not well studied. Our analysis suggests that the c-FLIP may be involved in the ZIKV-induced eye effects.

## 5. Conclusions

Our study provides compelling evidence that the knockdown of the *c-Flip* significantly impacts the transcriptomic profile of the fetal heads, with a notable emphasis on the processes integral to embryonic and central nervous system development. Furthermore, the intricate network of genes associated with apoptosis and an anti-viral immune response suggests a multifaceted role for the *c-Flip* in orchestrating key molecular events during viral infection.

## Data Availability

The RNA-sequencing data from this study have been deposited in the NCBI GEO database (http://www.ncbi.nlm.nih.gov/geo; accessed on 10 October 2024) under accession number GSE254990, and the raw sequence data deposited at the NCBI SRA database (http://www.ncbi.nlm.nih.gov/sra; accessed on 30 August 2024) under accession number PRJNA1072959.

## Supplementary Materials

The following supporting information can be downloaded at: https://www.mdpi.com/article/10.3390/v16111712/s1, Supplementary Figure S1: The network of interactions between the genes affected by the knockdown of the *c-Flip* in the fetal heads. The genes associated with several enriched functional categories shown in Figure 2 and Figure 4 were used to reconstruct the networks of genetic, physical, co-expression, co-localization, shared protein domains, and pathway interactions. Supplementary Figure S2: The common and specific gene numbers among up-regulated DEGs of *c-Flip*^+/−^ fetal heads compared with the WT fetal heads of the fetuses delivered by PBS-treated WT dams (blue), down-regulated DEGs of the *c-Flip*^+/−^ fetal heads compared with the WT fetal heads of fetuses delivered by PBS-treated WT dams (red), up-regulated DEGs of *c-Flip*^+/−^ fetal heads compared with WT fetal heads of fetuses delivered by ZIKV-infected WT dams (green) and down-regulated DEGs of *c-Flip*^+/−^ fetal heads compared with WT fetal heads of fetuses delivered by ZIKV-infected WT dams (pink). Supplementary Table S1: Statistics of the Illumina RNA-Seq reads in the WT and *c-Flip*^+/−^ fetal heads libraries that were mapped to the mice reference genome. Supplementary Table S2: The primers used to validate the hub genes in this research were listed below.
