# Supplementary material for: RNA-Seq Reveals Transcriptome Changes Following Zika Virus Infection in Fetal Brains in c-Flip Knockdown Mice
Source: Viruses. 2024 Oct 31;16(11):1712. doi: 10.3390/v16111712 (PMC11599063; doi:10.3390/v16111712)
Supplement: Supplementary file 1 [file viruses-16-01712-s001.zip › viruses-3246558-supplementary.pptx]

## Slide 1
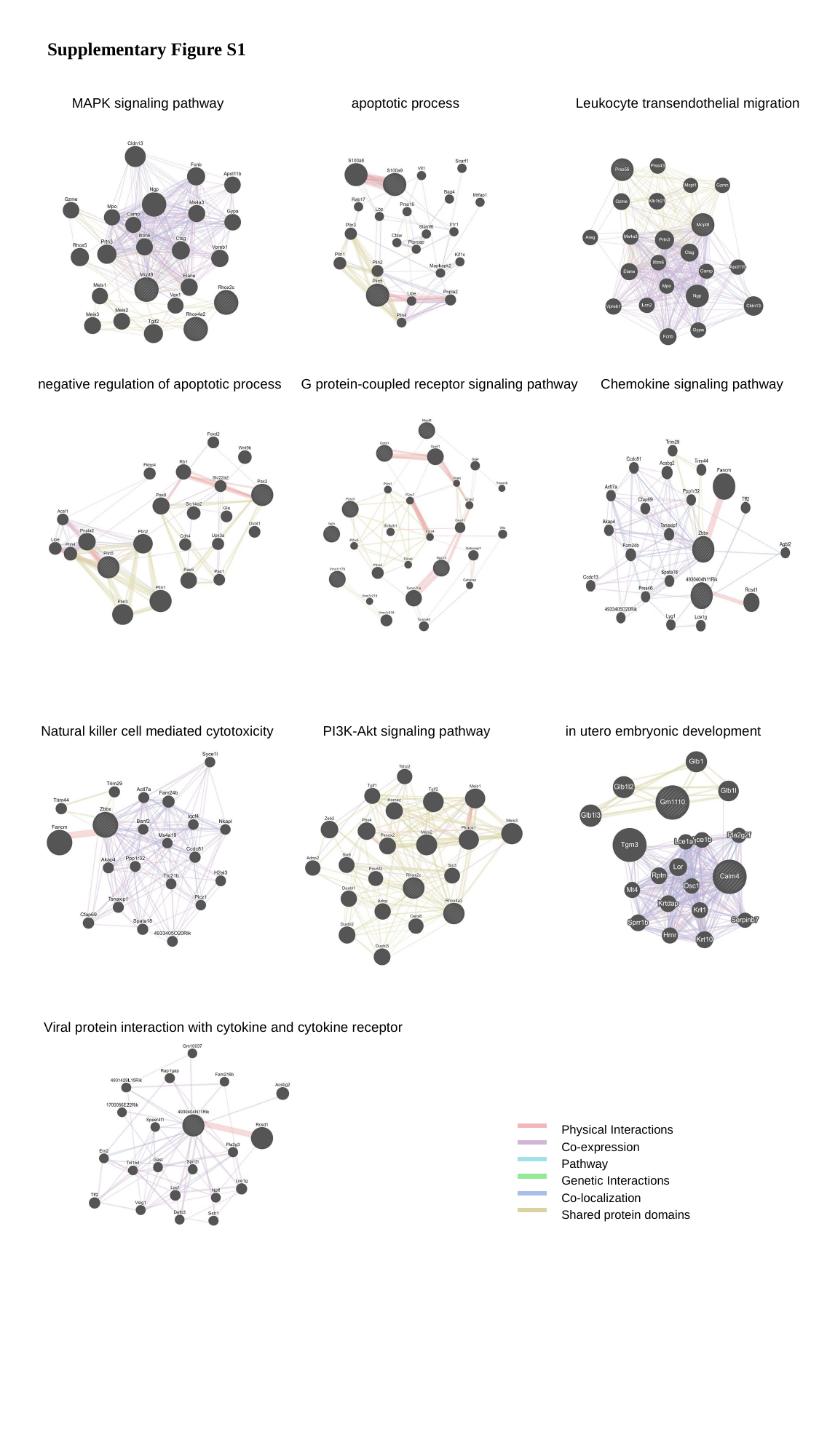

Supplementary Figure S1
MAPK signaling pathway
apoptotic process
Leukocyte transendothelial migration
negative regulation of apoptotic process
G protein-coupled receptor signaling pathway
Chemokine signaling pathway
PI3K-Akt signaling pathway
in utero embryonic development
Natural killer cell mediated cytotoxicity
Viral protein interaction with cytokine and cytokine receptor
Physical Interactions
Co-expression
Pathway
Genetic Interactions
Co-localization
Shared protein domains

## Slide 2
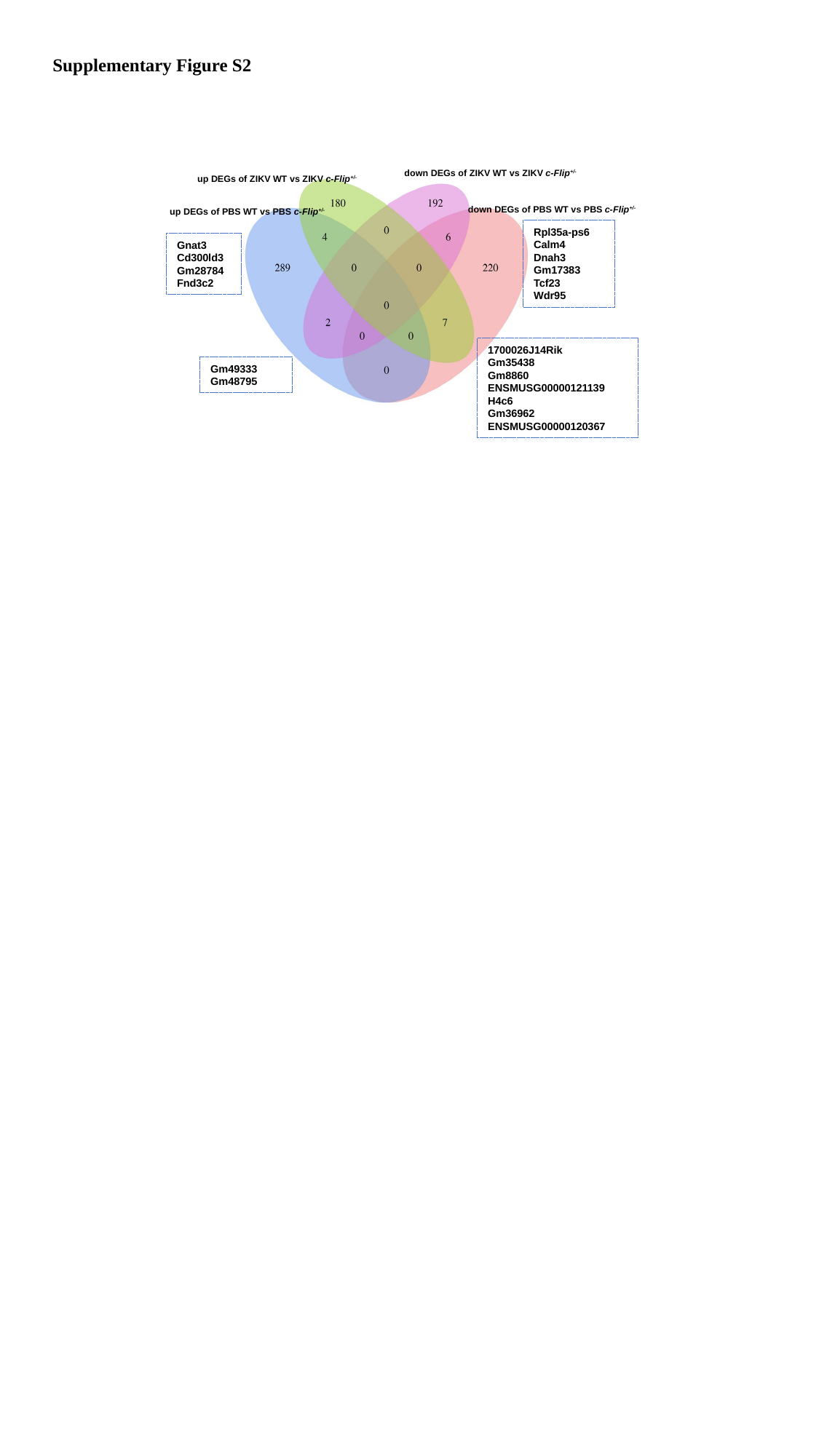

Supplementary Figure S2
down DEGs of ZIKV WT vs ZIKV c-Flip+/-
up DEGs of ZIKV WT vs ZIKV c-Flip+/-
down DEGs of PBS WT vs PBS c-Flip+/-
up DEGs of PBS WT vs PBS c-Flip+/-
Rpl35a-ps6
Calm4
Dnah3
Gm17383
Tcf23
Wdr95
Gnat3
Cd300ld3
Gm28784
Fnd3c2
1700026J14Rik
Gm35438
Gm8860
ENSMUSG00000121139
H4c6
Gm36962
ENSMUSG00000120367
Gm49333
Gm48795

## Slide 3
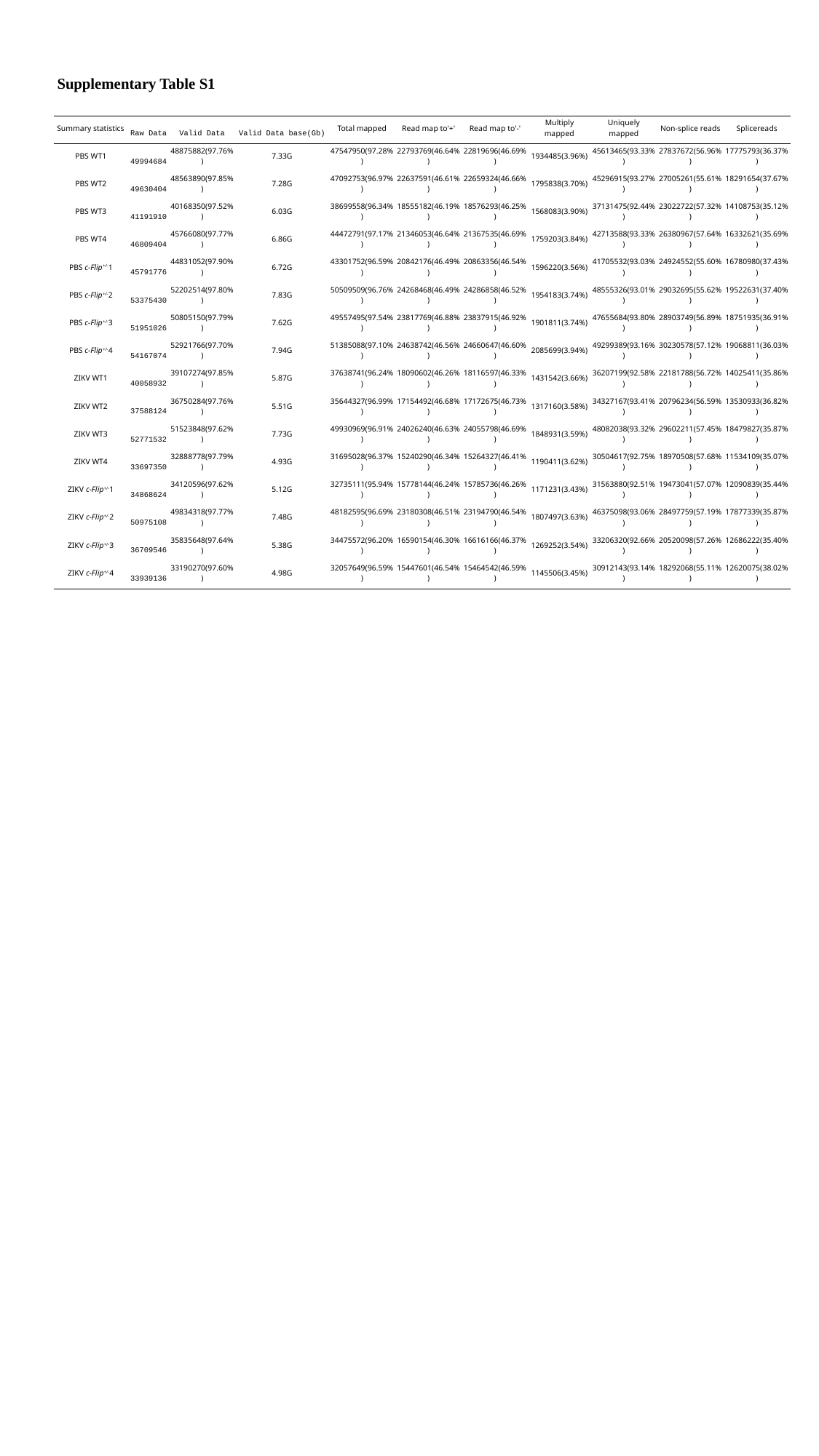

Supplementary Table S1
| Summary statistics | Raw Data | Valid Data | Valid Data base(Gb) | Total mapped | Read map to'+' | Read map to'-' | Multiply mapped | Uniquely mapped | Non-splice reads | Splicereads |
| --- | --- | --- | --- | --- | --- | --- | --- | --- | --- | --- |
| PBS WT1 | 49994684 | 48875882(97.76%) | 7.33G | 47547950(97.28%) | 22793769(46.64%) | 22819696(46.69%) | 1934485(3.96%) | 45613465(93.33%) | 27837672(56.96%) | 17775793(36.37%) |
| PBS WT2 | 49630404 | 48563890(97.85%) | 7.28G | 47092753(96.97%) | 22637591(46.61%) | 22659324(46.66%) | 1795838(3.70%) | 45296915(93.27%) | 27005261(55.61%) | 18291654(37.67%) |
| PBS WT3 | 41191910 | 40168350(97.52%) | 6.03G | 38699558(96.34%) | 18555182(46.19%) | 18576293(46.25%) | 1568083(3.90%) | 37131475(92.44%) | 23022722(57.32%) | 14108753(35.12%) |
| PBS WT4 | 46809404 | 45766080(97.77%) | 6.86G | 44472791(97.17%) | 21346053(46.64%) | 21367535(46.69%) | 1759203(3.84%) | 42713588(93.33%) | 26380967(57.64%) | 16332621(35.69%) |
| PBS c-Flip+/-1 | 45791776 | 44831052(97.90%) | 6.72G | 43301752(96.59%) | 20842176(46.49%) | 20863356(46.54%) | 1596220(3.56%) | 41705532(93.03%) | 24924552(55.60%) | 16780980(37.43%) |
| PBS c-Flip+/-2 | 53375430 | 52202514(97.80%) | 7.83G | 50509509(96.76%) | 24268468(46.49%) | 24286858(46.52%) | 1954183(3.74%) | 48555326(93.01%) | 29032695(55.62%) | 19522631(37.40%) |
| PBS c-Flip+/-3 | 51951026 | 50805150(97.79%) | 7.62G | 49557495(97.54%) | 23817769(46.88%) | 23837915(46.92%) | 1901811(3.74%) | 47655684(93.80%) | 28903749(56.89%) | 18751935(36.91%) |
| PBS c-Flip+/-4 | 54167074 | 52921766(97.70%) | 7.94G | 51385088(97.10%) | 24638742(46.56%) | 24660647(46.60%) | 2085699(3.94%) | 49299389(93.16%) | 30230578(57.12%) | 19068811(36.03%) |
| ZIKV WT1 | 40058932 | 39107274(97.85%) | 5.87G | 37638741(96.24%) | 18090602(46.26%) | 18116597(46.33%) | 1431542(3.66%) | 36207199(92.58%) | 22181788(56.72%) | 14025411(35.86%) |
| ZIKV WT2 | 37588124 | 36750284(97.76%) | 5.51G | 35644327(96.99%) | 17154492(46.68%) | 17172675(46.73%) | 1317160(3.58%) | 34327167(93.41%) | 20796234(56.59%) | 13530933(36.82%) |
| ZIKV WT3 | 52771532 | 51523848(97.62%) | 7.73G | 49930969(96.91%) | 24026240(46.63%) | 24055798(46.69%) | 1848931(3.59%) | 48082038(93.32%) | 29602211(57.45%) | 18479827(35.87%) |
| ZIKV WT4 | 33697350 | 32888778(97.79%) | 4.93G | 31695028(96.37%) | 15240290(46.34%) | 15264327(46.41%) | 1190411(3.62%) | 30504617(92.75%) | 18970508(57.68%) | 11534109(35.07%) |
| ZIKV c-Flip+/-1 | 34868624 | 34120596(97.62%) | 5.12G | 32735111(95.94%) | 15778144(46.24%) | 15785736(46.26%) | 1171231(3.43%) | 31563880(92.51%) | 19473041(57.07%) | 12090839(35.44%) |
| ZIKV c-Flip+/-2 | 50975108 | 49834318(97.77%) | 7.48G | 48182595(96.69%) | 23180308(46.51%) | 23194790(46.54%) | 1807497(3.63%) | 46375098(93.06%) | 28497759(57.19%) | 17877339(35.87%) |
| ZIKV c-Flip+/-3 | 36709546 | 35835648(97.64%) | 5.38G | 34475572(96.20%) | 16590154(46.30%) | 16616166(46.37%) | 1269252(3.54%) | 33206320(92.66%) | 20520098(57.26%) | 12686222(35.40%) |
| ZIKV c-Flip+/-4 | 33939136 | 33190270(97.60%) | 4.98G | 32057649(96.59%) | 15447601(46.54%) | 15464542(46.59%) | 1145506(3.45%) | 30912143(93.14%) | 18292068(55.11%) | 12620075(38.02%) |

## Slide 4
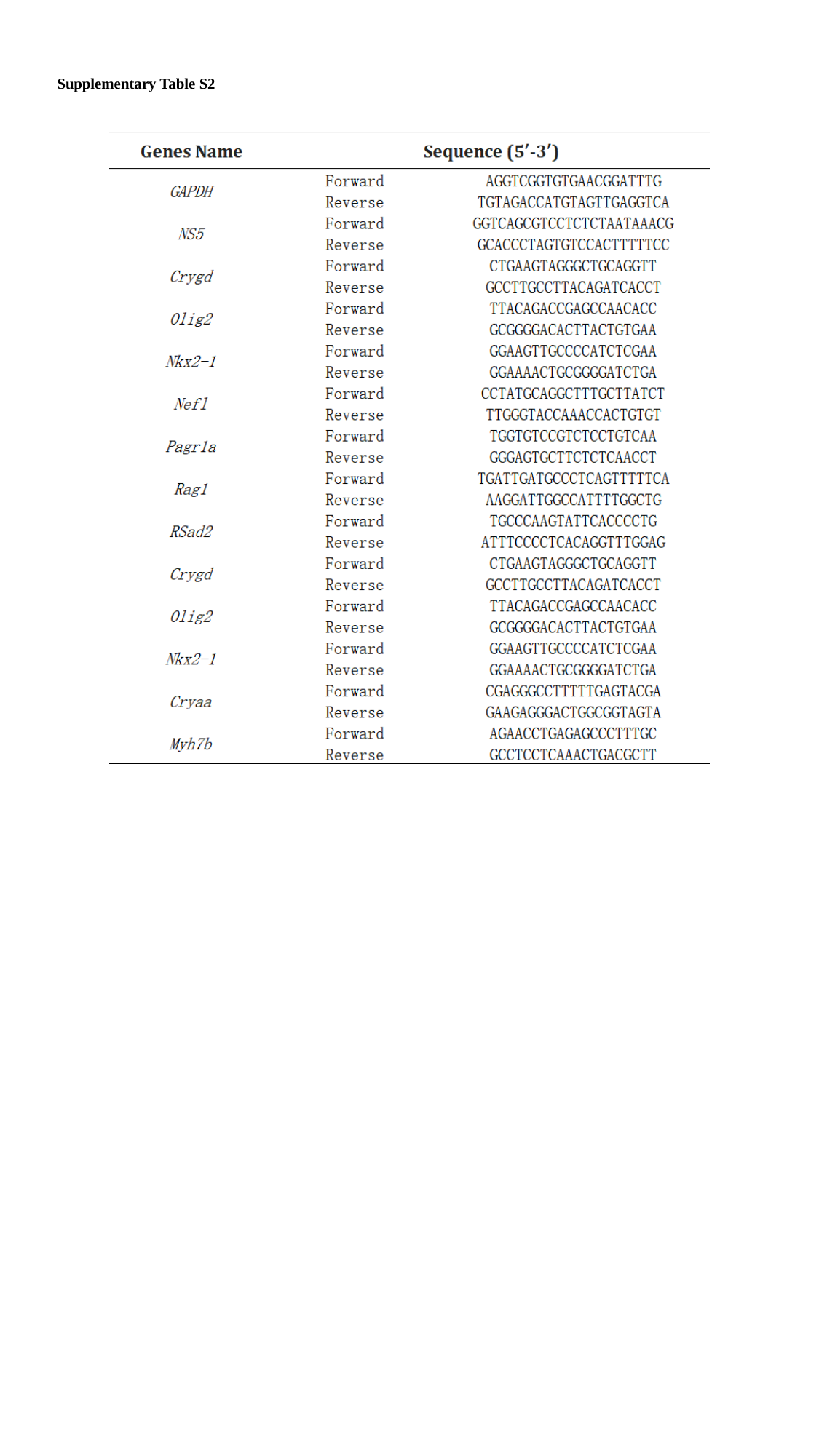

Supplementary Table S2
